# Supplementary material for: Hexameric NuMA:LGN structures promote multivalent interactions required for planar epithelial divisions
Source: Nat Commun. 2019 May 17;10:2208. doi: 10.1038/s41467-019-09999-w (PMC6525239; doi:10.1038/s41467-019-09999-w)
Supplement: Supplementary file 1 — Supplementary Information [file 41467_2019_9999_MOESM1_ESM.pdf]

**Hexameric NuMA:LGN structures promote multivalent interactions required for planar epithelial divisions.**

Pirovano et al.

## SUPPLEMENTAL INFORMATION

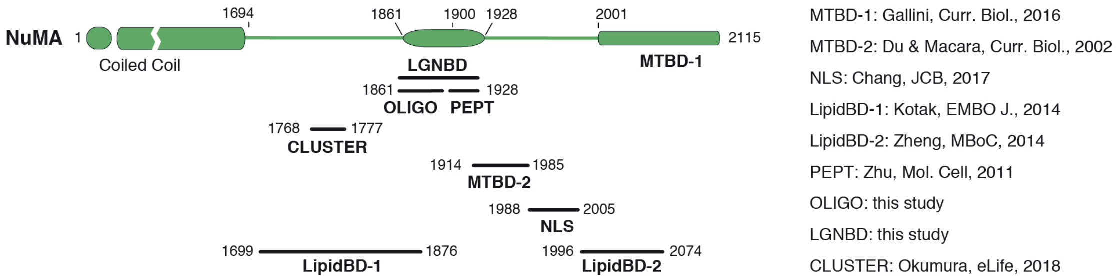


**Supplementary Figure 1. Functions of the C-terminal cargo-binding region of NuMA.** Scheme of the diverse function coded by the C-terminal region of NuMA based on the literature and the current study.

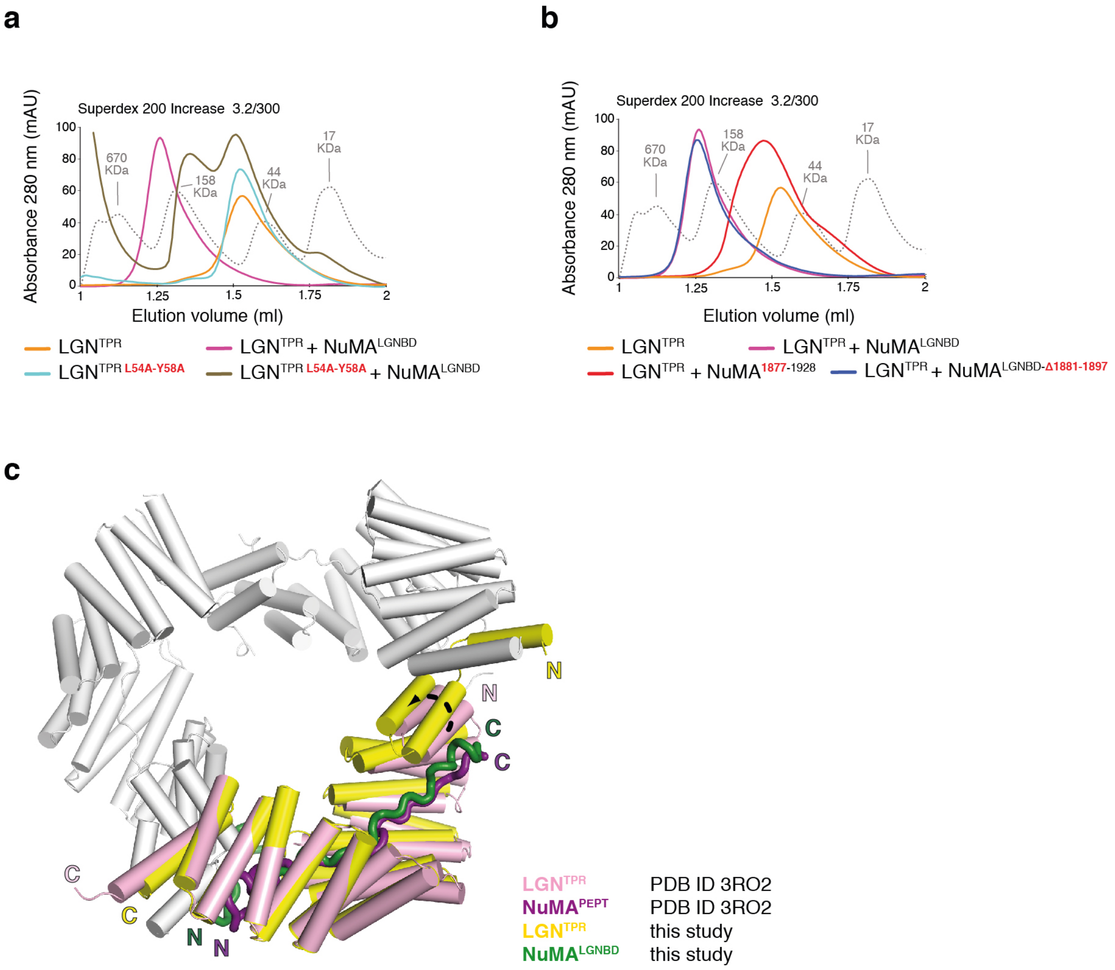


**Supplementary Figure 2. Analysis of the molecular determinants for the NuMA/LGN$^{TPR}$ hexamers formation.** (**a**) Analytical SEC elution profile of NuMA$^{LGNBD}$ in complex with LGN$^{TPR}$ wild-type (purple trace) or carrying the double mutation in L54A-Y58A in the TPR-2 helix-A facing the NuMA oligomerizing stretch (olive trace, Fig. 2f). LGN$^{TPR}$ mutations at the interface with NuMA$^{OLIGO}$ result in lower molecular weight complexes. (**b**) SEC elution profiles of LGN$^{TPR}$ in complex with truncations of NuMA$^{LGNBD}$. N-terminal truncation of the NuMA$^{LGNBD}$ to residue 1877 results in a late eluting complex, consistent with a 1:1 stoichiometry (red trace). Conversely, LGN$^{TPR}$ bound to NuMA$^{LGNBD}$ (purple trace) or to NuMA$^{LGNBD\text{-}\Delta 1881\text{-}1897}$ (blue trace) elute in the same fraction, indicating that NuMA residues 1881-1897 are dispensable for oligomerization. This finding suggests that NuMA residues 1881-1897 are not stabilized by contacting LGN$^{TPR}$, and is consistent with the missing electron

density for this region that we noticed in the crystal structure. In both panels, the elution profile of globular markers is reported in a dashed gray line. (**c**) Structural comparison of $LGN^{TPR}$ in complex with $NuMA^{PEPT}$ (PDB ID 3RO2) or with $NuMA^{LGNBD}$ (this study). The two structures were superposed aligning LGN residues 234 to 367, including TPR-6 to TPR-8 and the capping helix. LGN molecules are depicted as cylindrical helices (pink for 3RO2, yellow and white for this study), and NuMA chains are depicted as ribbon (purple for 3RO2, green for this study).

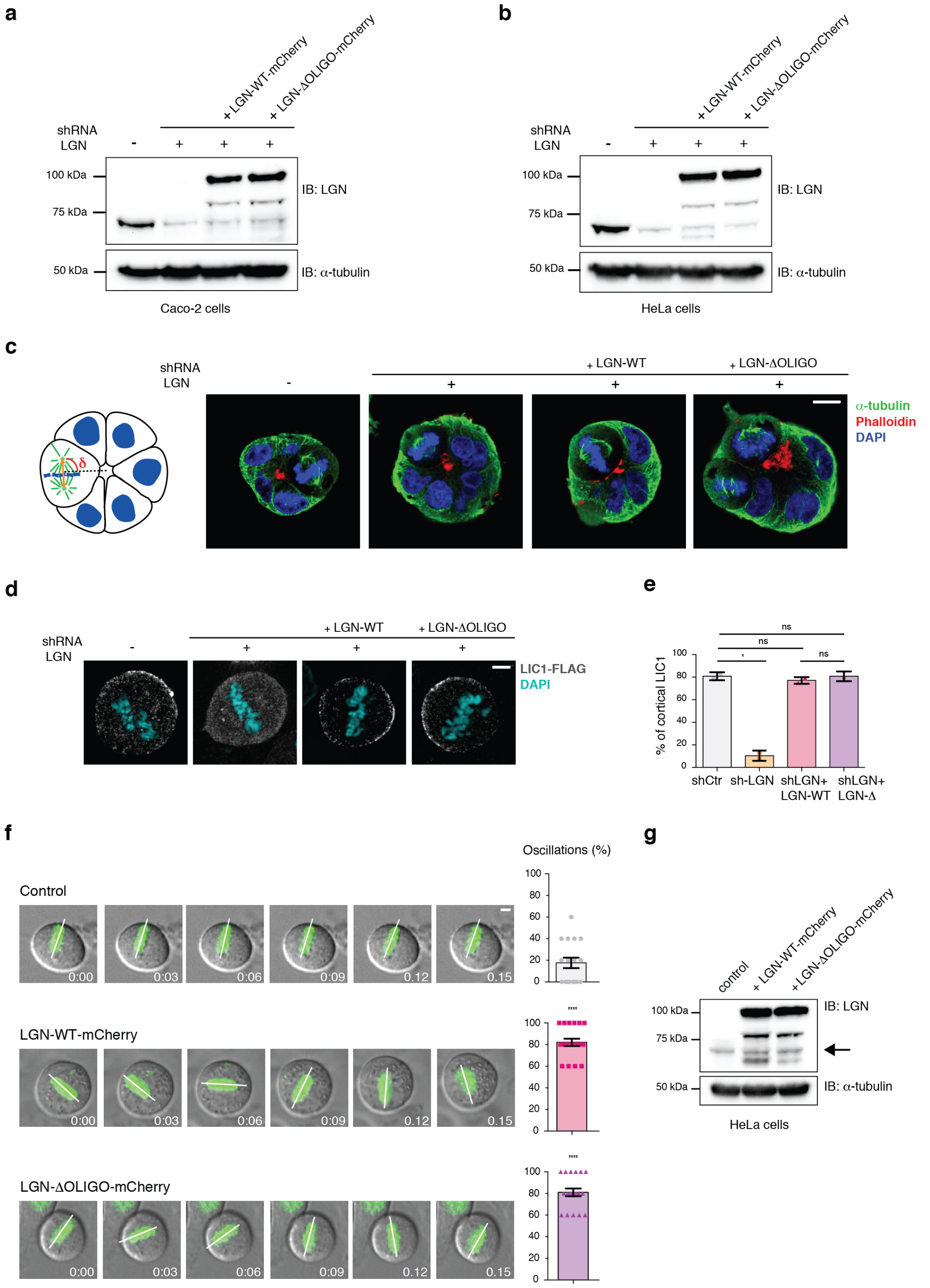


**Supplementary Figure 3. NuMA/LGN oligomerization is required for oriented planar cell divisions, but not for dynein cortical recruitment.** (**a-b**) Immunoblot of mitotic lysates of Caco-2 and HeLa cell lines stably depleted of LGN and expressing LGN-WT-mCherry or

LGN-ΔOLIGO-mCherry. α-tubulin was used as loading control. (**c**) Left: Diagram depicting spindle angle orientation during physiological planar divisions of growing Caco-2 cysts, and the spindle axis angle δ measured. Right: Confocal sections of Caco-2 cysts grown from cells wild-type or depleted of endogenous LGN and expressing the LGN wild-type-mCherry or LGN-ΔOLIGO-mCherry rescue constructs. Cysts were stained with α-tubulin to visualize the mitotic spindle (green), Phalloidin to visualize the apical lumen (red), and DAPI to image DNA (blue). (**d**) Confocal sections of HeLa cells stably depleted of endogenous LGN, stably expressing mCherry C-terminally-tagged LGN-WT or LGN-ΔOLIGO, and transfected with FLAG-tagged dynein light-intermediate chain 1 (LIC1). Mitotic cells were stained with anti-FLAG antibody to visualize LIC1 (white) and DAPI to visualize the metaphase plate (cyan). (**e**) Analysis of FLAG signal at the cortex in metaphase HeLa cells expressing 3xFLAG-LIC1. Per each condition shown in panel d, the mean percentage of cells displaying cortical FLAG signal is plotted ± SD (with $n > 32$ from two independent experiments). In the plot, LGN-Δ stands for LGN-ΔOLIGO. * $p < 0.05$ by Fisher's exact test, *ns* not-significant. (**f**) Analysis of spindle oscillation in HeLa cells over-expressing LGN-WT and LGN-ΔOLIGO. Images from time lapse microscopy of metaphase HeLa cells stably expressing GFP-H2B transfected with LGN-WT-mCherry or LGN-ΔOLIGO-mCherry. The GFP-H2B signal is overlaid with DIC, the position of the metaphase plate is marked with a white line. About 10 cells per condition were filmed in 2 independent experiments, taking frames every 3 minutes. The extent of oscillation was calculated considering the frequency at which the metaphase plate rotates more than 10º between two consecutive frames, and plotted in bar graphs on the right along with the SD. A two-tailed Student's test was applied to assess the statistical relevance between transfected conditions and non-transfected control cells. **** $p < 0.0001$. (**g**) Immuno-blot showing the levels of ectopically expressed LGN-mCherry proteins in mitotic HeLa cell lysates. α-tubulin is shown as loading control. The band of endogenous LGN is indicated with a black arrow. Scale bars 5 μm for HeLa cells and 10 μm for Caco-2 cysts.

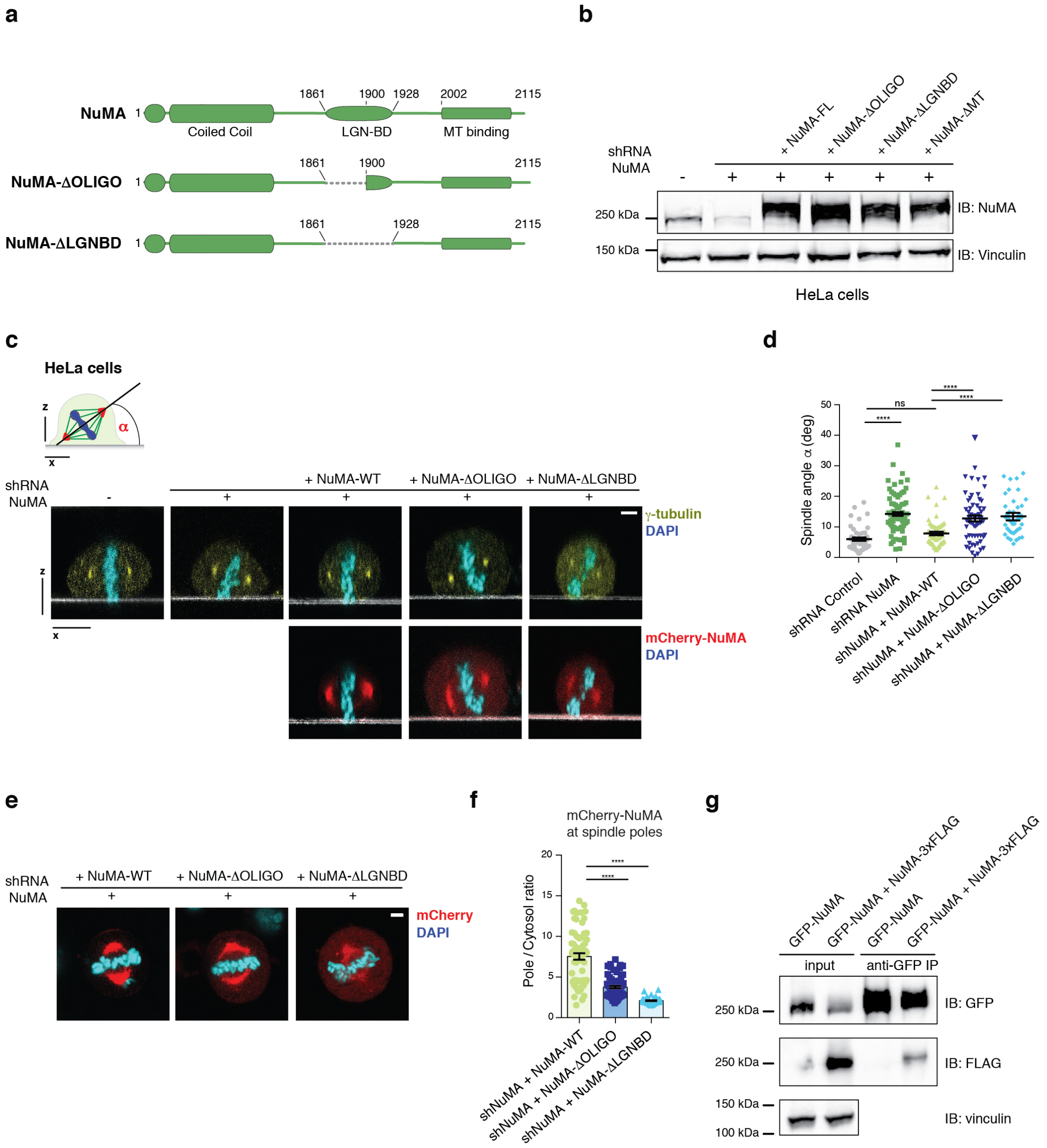


**Supplementary Figure 4. Oligomerization-deficient NuMA mutants cannot sustain spindle orientation nor correct spindle pole organization.** (**a**) Schematic representation of the domain structure of NuMA wild-type and mutants. Dashed lines indicate fragments of NuMA deleted to generate the indicated rescue constructs. (**b**) Immunoblot of mitotic lysates of HeLa cells stably depleted of NuMA and transfected with the mCherry-NuMA rescue constructs indicated, i.e. NuMA wild-type, NuMA-ΔOLIGO, NuMA-ΔLGNBD, and NuMA-ΔMT. Vinculin was used as loading control. (**c**) Confocal x-z sections of metaphase HeLa cells depleted of endogenous NuMA and transfected with the indicated mCherry-tagged NuMA

rescue constructs. Cells were stained with γ-tubulin (yellow) and DAPI (blue). (**d**) Dot-plot illustrating the distribution of the spindle axis angles for the experiments of panel c. Means ± SEM are shown for four independent experiments, with n=51 for control cells (expressing a scrambled shRNA), n=71 for NuMA shRNA expressing cells, n=56 for NuMA-depleted cells transfected with NuMA-ΔOLIGO, and n=45 for NuMA-depleted cells transfected with NuMA-ΔLGNBD. For statistics, the Krustal-Wallis test was applied, **** indicates $p < 0.0001$. (**e**) Confocal images of metaphase HeLa cells stably depleted of NuMA and expressing mCherry-tagged NuMA rescue constructs. The metaphase plate is visualized by DAPI staining (blue). (**f**) Quantification of the mCherry fluorescent signal at the poles (per each cell, one spindle pole in focus was considered), with histograms representing the poles-to-cytoplasm fluorescent ratio (see Methods for details). Means ± SEM are shown for three independent experiments, with n>60. The Krustal-Wallis test was applied. **** indicates $p < 0.0001$. (**g**) Full-length NuMA self-assemble. HEK293T cells were transiently co-transfected with plasmids expressing GFP-NuMA and NuMA-3xFLAG. After 48 hours, mitotic lysates were immunoprecipitated (IP) with GFP-antibody conjugated to sepharose beads, and immuno-blotted (IB) with the indicated antibodies. Cells transfected only with GFP-NuMA were used as a specificity control. Scale bars, 5 μm.

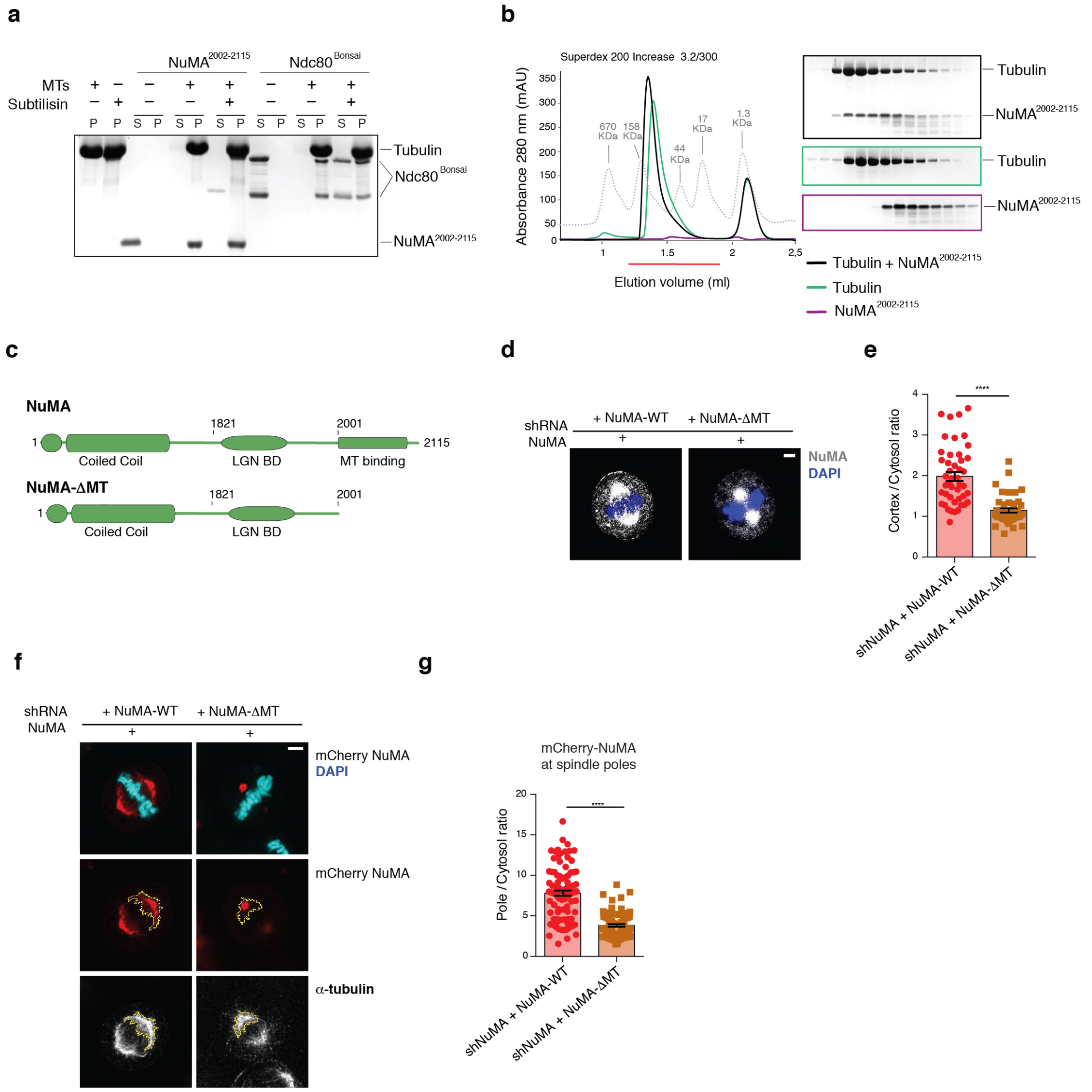


**Supplementary Figure 5. Characterization of the microtubule-binding activity of NuMA.** (**a**) Co-sedimentation assays performed with 9 µM of paclitaxel-stabilized microtubules with or without tubulin tails and 5 µM of NuMA$^{2002-2115}$, encompassing the MT-binding domain. The supernatant (S) and pellet (P) fractions were analysed on a Coomassie-stained SDS-PAGE. Ndc80$^{Bonsai}$ was used as a control of tail-dependent MT-binding. The solubility of NuMA$^{2002-2115}$ and Ndc80$^{Bonsai}$ in the absence of microtubules was also tested. The experiment shows that NuMA$^{2002-2115}$ binds to the MTs lattice regardless of the tubulin tails. (**b**) SEC elution profile of αβ-tubulin hetero-dimers in complex with NuMA$^{2002-2115}$ (black trace). Individual SEC runs of αβ-tubulin (green trace) and NuMA$^{2002-2115}$ (purple trace) are also shown. The elution of

globular markers is indicated as a dashed gray dotted line. Peak fractions indicated by the horizontal red bar were analysed by Coomassie-stained Tris-Tricine-SDS-PAGE. (**c**) Schematic representation of the domain structure of NuMA wild-type and NuMA-ΔMT according to Gallini[1]. (**d**) HeLa cells depleted of endogenous NuMA and transfected with mCherry-tagged NuMA wild-type or mCherry-NuMA-ΔMT were synchronized in metaphase and stained for NuMA (white) and DAPI (blue). (**e**) Quantification of the cortex-to-cytoplasm ratio of the NuMA-antibody signal for the experiments presented in d. Histograms with means ± SEM are shown for three independent experiments, with $n > 45$. The Mann-Whitney test was applied. **** indicates $p < 0.0001$. (**f**) Confocal sections of metaphase HeLa cells depleted of endogenous NuMA and transfected with mCherry-NuMA wild-type or mCherry-NuMA-ΔMT. Cells were stained with α-tubulin to visualize the spindle (white) and with DAPI to visualize the DNA (blue). The tubulin signal was used to draw a mask (yellow line) to quantify the mCherry-NuMA signal (see Methods for the details). (**g**) Quantification of mCherry-NuMA at the poles, with histogram representing the poles-to-cytoplasm fluorescent ratio. Means ± SEM are shown for four independent experiments with $n>66$. **** $p < 0.0001$ by Mann-Whitney test. Scale bars, 5 μm.

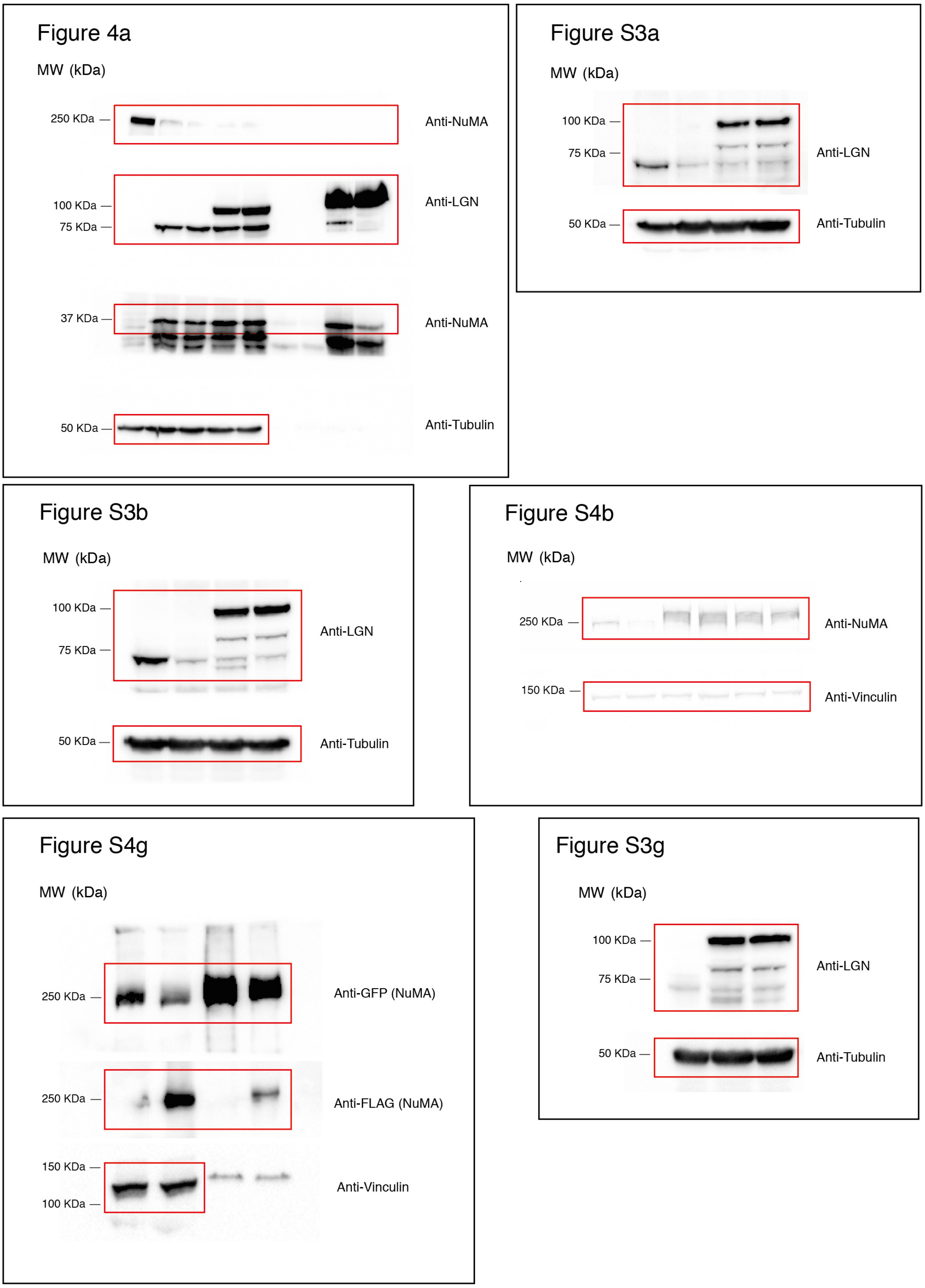


**Supplementary Figure 6. Uncropped images of SDS-PAGES presented in the main and supplementary figures.**

## Supplementary Table 1. PCR primers

| PRIMER NAME | PRIMER SEQUENCE | VECTOR NAME |
|---|---|---|
| **BIOCHEMISTRY** | | |
| LGN-1_FOR | GCGCGCGGATCCATGAGAGAAGACCATTCT | pGEX-6P1-GST-LGN1-409, GST-LGN1-350 |
| LGN-7_FOR | GCGCGCGGATCCATGTTTCATGTTCGTTACAGA | pGEX-6P1-GST-LGN7-367 |
| LGN-13_FOR | GCGCGCGGATCCATGGAAGCTTCTTGCCTA | pGEX-6P1-GST-LGN13-409, GST-LGN13-350 |
| LGN-350_REV | GCGCGCGTCGACTTAATCCCCAACCTCTCTTGA | pGEX-6P1-GST-LGN1-350, GST-LGN13-350 |
| LGN-409_REV | GCGCGCGTCGACTTACTTCATAAGTTCCATATT | pGEX-6P1-GST-LGN1-409, GST-LGN13-409 |
| LGN-368_REV | GCGCGCGTCGACTTAAAGAACCATTTGAAGGTC | pGEX-6P1-GST-LGN7-367 |
| LGN-L54A-Y58A_FOR | GAAGACCTAAAAACAGCTAGCGCTATTGCCAGCCAGTTGGGCAATG | pGEX-6P1-GST-LGN-L54AY58A |
| LGN-L54A-Y58A_REV | CATTGCCCAACTGGCTGGCAATAGCGCTAGCTGTTTTTAGGTCTTC | pGEX-6P1-GST-LGN-L54AY58A |
| NuMA-1592_FOR | GCGCGCCCATGGGTCAGCTGAATGAACTGCAAG | pEMT14-His-NuMA1592-1694, NuMA1592-2001 |
| NuMA-1821_FOR | GCGCGCCCATGGGTAAGAAGCTAGATGTGGAA | pETM14-GST-NuMA1821-1928, NuMA1821-2001 |
| NuMA-1861_FOR | GCGCGCGGATCCATGGGTTCTCCCGATTATGGC | pGEX-6P1-GST-NuMA1861-1928 |
| NuMA-1877_FOR | GCGCGCGGATCCATGCGCCCCACCACTCGCAGT | pGEX-6P1-GST-NuMA1877-1928, NuMA 1877-2001 |
| NuMA-1900_FOR | GCGCGCGGATCCATGAACAGCTTCTACATGGGC | pETM14-GST-NuMA1900-1928 |
| NuMA-2002_FOR | GCGCGCGGATCCGAGTCTAAGAAGGCCACC | pETM14-His-NuMA2002-2115 |
| NuMA-1694_REV | GCGCGCGTCGACTTACAGCTGCTGGTCAGC | pETM14-His-NuMA1592-1694 |
| NuMA-1928_REV | GCGCGCGTCGACTTATCGATTGCGCTGCTGCAG | pGEX-6P1-GST-NuMA1861-1928, NuMA1877-1928<br>pEMT14-GST-NuMA1821-1928, NuMA1900-1928 |
| NuMA-2001_REV | GCGCGCGTCGACTTAAGGAGTTCCAGGGCCCTG | pETM14-His-NuMA1592-001, GST-NuMA1821-2001<br>GST-NuMA1877-2001 |
| NuMA-2115_REV | GCGCGCGTCGACTTAGTGCTTTGCCTTGCCCTT | pETM14-His-NuMA2002-2115 |
| NuMA-1592_FOR | ACCGGCTCTACCGGCTCTAAGAAGCTAGATGTGGAAGAGCC | pETM14-His-NuMA1592-2001-TGS |
| NuMA-2001_REV | AGAGCCGGTCTGCTGGTCTGCATGGGCAAC | pETM14-His-NuMA1592-2001-TGS |
| NuMA-1897_FOR | ACCGGCTCTGGAAGGAACAGCTTCTACATGGGCACTTGC | pGEX-6P1-GST-NuMAΔ1881-1897 |
| NuMA-1881_REV | AGAGCCGGTAGTGGTGGGGCGGTAGCCAGGC | pGEX-6P1-GST-NuMAΔ1881-1897 |
| **CELL BIOLOGY** | | |
| LGN-1_FOR | GCGCGCGCTAGCACCATGAGAGAAGACCATTCTTTTC | pCDH-LGN-WT-mCherry |
| LGN-13_FOR | GCGCGCGCTAGCACCATGGAAGCTTCTTGCCTA | pCDH-LGN-ΔOLIGO-mCherry |
| LGN-677_REV | GCGCGCGGATCCATGGTCTGCCGATTTTTTC | pCDH-LGN-WT/ΔOLIGO-mCherry |
| LGN-368_FOR | GGTCTGAGCTACAGCACAAATAAC | pCDH-LGN-ΔOLIGO-mCherry |
| LGN-350_REV | ATCCCCAACCTCTCTTGAAATTTC | pCDH-LGN-ΔOLIGO-mCherry |
| NuMA-stop2002_FOR | GGCCCGGGAACCCCCTAGTCCAAGAAGGCTACC | pCDH-mCherry-NuMA-ΔMT |
| NuMA-stop2002_REV | GGTAGCCTTCTTGGACTAGGGGGTTCCCGGGCC | pCDH-mCherry-NuMA-ΔMT |
| NuMA-1821_FOR | GCGCGCGGATCCAAGAAGCTAGATGTGGAA | pCDH-NuMA-1821-2215 |
| NuMA-1901_FOR | TCCTTCTACATGGGAACCTGCCAG | pCDH-mCherry-NuMA-ΔOLIGO, GFP-NuMA-ΔOLIGO-GoLoco |
| NuMA-1928_FOR | CGTGTGTGCCCCCCTCACCTCAAG | pCDH-mCherry-NuMA-ΔLGN, GFP-NuMA-ΔLGN-GoLoco |
| NuMA-1861_REV | CAGACGAGCCAGGGACTGGGTAGA | pCDH-mCherry-NuMA-ΔOLIGO/ΔLGN |
| NuMA-2115_REV | GCGCGCGGCCGCCCCTTAGTGCTTTGCCTTGCCC | pCDH-NuMA-1821-2215 |
| LIC1-1_FOR | GCGCGCGCTAGCACCATGGCGGCCGTGGGG | pCDH-FLAG-LIC1 |
| LIC1-523_REV | GCGCGCGGATCCAGAAGCTTCTCCTTCCGTAGGAGA | pCDH-FLAG-LIC1 |
| shLGN_FOR | GGATGTAGTGGGAAACAATTTCAAGAGAATTGTTTCCCACTACATCCTTTTTTC | pll3.7-GFP-shLGN |
| shLGN_REV | TCGAGAAAAAAGGATGTAGTGGGAAACAATTCTCTTGAAATTGTTTCCCACTACATCC | pll3.7-GFP-shLGN |
| **IMMUNOPRECIPITATION** | | |
| LGN-1_FOR | GCGCGCGGATCCATGAGAGAAGACCATTCT | pEGFP-GFP-LGN-WT |
| LGN-13_FOR | GCGCGCGGATCCATGGAAGCTTCTTGCCTA | pEGFP-GFP-LGN-ΔOLIGO |
| LGN-677_REV | GCGCGCGTCGACTTAATGGTCTGCCGATTTTTT | pEGFP-GFP-LGN-WT/ΔOLIGO |
| FLAG_FOR | GCGCGCGGATCCGATTATAAGGATGACGATGACAAAG | pCDH-NuMA-3XFLAG, LGN-WT/ΔOLIGO-3XFLAG |
| FLAG_REV | GCGCGCGGCCGCGACTGATAGTGACCTGTTCG | pCDH-NuMA-3XFLAG, LGN-WT/ΔOLIGO-3XFLAG |

## References


1. Gallini, S. et al. NuMA Phosphorylation by Aurora-A Orchestrates Spindle Orientation. *Current biology : CB* (2016).
